# All-inkjet-printed thin-film transistors: manufacturing process reliability by root cause analysis

**DOI:** 10.1038/srep33490

**Published:** 2016-09-21

**Authors:** Enrico Sowade, Eloi Ramon, Kalyan Yoti Mitra, Carme Martínez-Domingo, Marta Pedró, Jofre Pallarès, Fausta Loffredo, Fulvia Villani, Henrique L. Gomes, Lluís Terés, Reinhard R. Baumann

**Affiliations:** 1 Technische Universität Chemnitz (TUC), Digital Printing and Imaging Technology, 09126 Chemnitz, Germany; 2 Institut de Microelectrònica de Barcelona, IMB-CNM (CSIC), 08193 Bellaterra, Spain; 3 Italian National Agency for New Technologies, Energy and Sustainable Economic Development (ENEA), Portici Research Center, 80055 Portici (Naples), Italy; 4 Universidade do Algarve, FCT, Campus de Gambelas, 8000-139 Faro, Portugal; 5 Instituto de Telecomunicações (IT), Organic Electronics-Lx, 1049-001 Lisboa, Portugal; 6 Fraunhofer Institute for Electronic Nanosystems (ENAS), Department of Printed Functionalities, 09126 Chemnitz, Germany

## Abstract

We report on the detailed electrical investigation of all-inkjet-printed thin-film transistor (TFT) arrays focusing on TFT failures and their origins. The TFT arrays were manufactured on flexible polymer substrates in ambient condition without the need for cleanroom environment or inert atmosphere and at a maximum temperature of 150 °C. Alternative manufacturing processes for electronic devices such as inkjet printing suffer from lower accuracy compared to traditional microelectronic manufacturing methods. Furthermore, usually printing methods do not allow the manufacturing of electronic devices with high yield (high number of functional devices). In general, the manufacturing yield is much lower compared to the established conventional manufacturing methods based on lithography. Thus, the focus of this contribution is set on a comprehensive analysis of defective TFTs printed by inkjet technology. Based on root cause analysis, we present the defects by developing failure categories and discuss the reasons for the defects. This procedure identifies failure origins and allows the optimization of the manufacturing resulting finally to a yield improvement.

Printing technologies are considered as promising approach for the manufacturing of novel flexible and large area electronics. Several different kinds of printed electronic devices have been demonstrated in literature such as resistors^1^,^2^, antennas^3^,^4^, capacitors^5^,^6^, diodes^7^,^8^, sensors^9^,^10^, photovoltaic cells^11^,^12^, displays^13^,^14^, batteries^15^, and thin-film transistors^16^,^17^ (TFTs). Despite of the huge efforts towards manufacturing process and device performance optimization, there are still rarely examples that show a sustainable and successful transfer to the industry. There might be multiple reasons for the limited success of printed electronics in industry so far. However, one important fact is the stability and reliability of the printing process with the new functional ink materials^18^. Printed electronics is usually compared with conventional lithography-based microelectronics. However, the processes used in conventional electronics are highly mature, well-established methods with exceptional accuracy, stability and reliability. Printing methods and especially inkjet printing can usually not compete in terms of these features with conventional silicon microelectronic technologies^19^. Printing has been initially developed to replicate visible information by deposition of colored liquids or pastes on e.g. paper-based substrates. Thus, the technology was designed to the human visual perception tolerating 100 μm misalignments of deposits to be still rated as high quality print^19^. Although printing methods have been exploited to deposit other liquids and pastes, e.g. containing particles or molecules with the functional properties for electronic applications, such as conductive, semiconducting or dielectric particles or molecules, they were not intentionally foreseen for these purposes. The adaption of the existing printing technologies and processes towards the deposition of functional layers and devices are still current topics of research and might have high relevance for the future industry of manufacturing. So far, there is no detailed and comprehensive study available focusing on the investigation of the process stability and reliability of printing electronic devices and the failure origins in the process.

Our contribution focuses on the failures appearing in inkjet-printed electronics based on the developed process chain for the manufacturing of all-inkjet-printed TFTs reported elsewhere^17^. These failures are responsible for defects limiting the yield, namely the number of devices functioning properly. Exemplarily, a very complex and the most frequently used electronic device – the TFT – was chosen allowing to address the multiple failure origins due to the usage of a multilayer stack employing different materials and printing parameters. Thus, the focus of this contribution is set on a comprehensive analysis of defective TFTs manufactured by inkjet printing which has not been addressed sufficiently in literature before. The developed process platform is dedicated to the manufacturing of a large number of all-inkjet-printed TFTs on flexible, DIN A4-sized PEN substrates allowing to perform a statistical relevant device characterization^17^. All deposition processes were carried out by using inkjet printing in ambient conditions and at low temperature. Statistical analysis of the TFTs using a semiautomatic characterization system enabled the determination of process yield and failure origins for defective devices. Optimization of the manufacturing process in terms of stability and reliability can be achieved by means of the assessment of the origin of failures.

## Results and Discussion

### Printing process development

**Figure 1:**
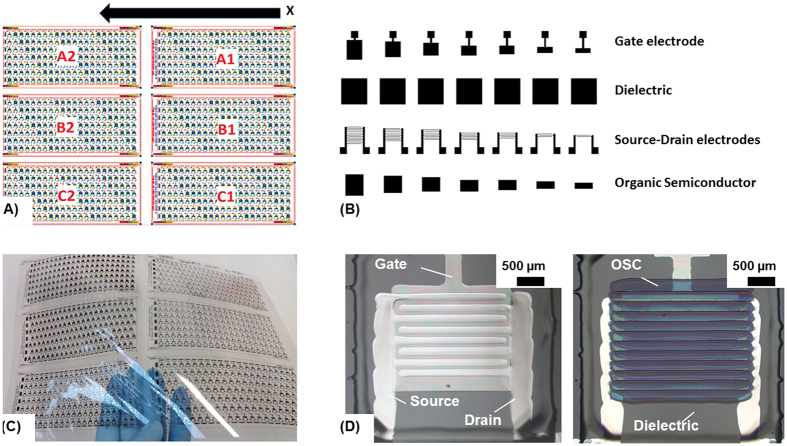
(**A**) Layout of the design for 924 TFTs arranged in six arrays each with 154 TFTs based on the BGBC architecture, (**B**) print pattern layout of the individual layers of the TFTs having different active areas by variation of the gate, S-D and OSC layer layout which results in the different W/L ratios; (**C**) all-inkjet-printed TFT array as designed in (**A**) on flexible PEN substrate and (**D**) microscopic images of TFTs with W/L of 140 showing the TFT layer stack with (right) and without (left) OSC.

All-inkjet-printed TFTs arrays were manufactured as described in materials and methods on DIN A4-sized PEN films. The scheme in Fig. 1A shows the printing origin marked with x and the printing direction for each layer. Thus, the printing starts with the array A1 line by line and finishes with array C2. Printing was performed unidirectional, so that the deposition takes place only in the indicated direction. This specific printing direction was mainly chosen to allow the deposition of the S-D electrodes along the printing transfer path resulting in well-defined line morphologies. Thus, any misalignment effects are avoided.

**Figure 2:**
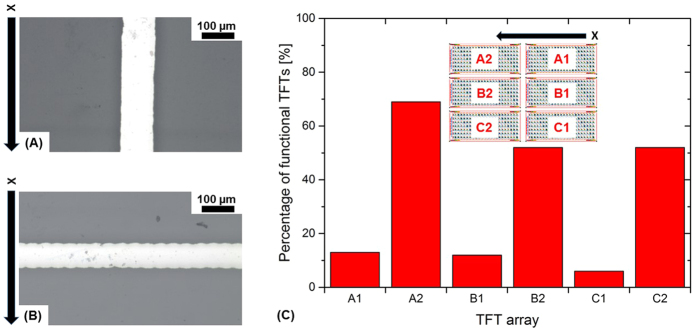
Inkjet-printed line with silver ink on PEN substrate deposited (**A**) along printing direction and (**B**) perpendicular to printing direction; (**C**) percentage of functional TFTs per TFT array (each array has 154 TFTs).

The influence of the printing direction on the morphology of the deposited electrode is depicted in Fig. 2A,B. Printing perpendicular to the finger electrode directions usually results in lower line width, lower edge sharpness and higher line thickness and roughness^20^. Therefore, the homogeneity is lower compared to the line morphology obtained by printing along the printing direction increasing the risk of device failures. The occurrence of the different line morphology as a function of printing direction is mainly due to the droplet jetting frequency. It is much higher perpendicular to the printing direction than along the printing as explained in our previous works^20^. Detailed studies about line formation were done by Soltman *et al*.^21^ demonstrating the importance of print resolution and the droplet ejection frequency on the morphology of the inkjet-printed lines.

The spacing between the S-D electrode fingers, finally referring to the channel length of the TFT, was optimized by printing several thousands of test layouts consisting of two straight lines printed next to each other with different distances. Thus, arrays of these test line patterns with different spatially distributed spacing were printed and electrically characterized to extract the most reliable and at the same time the smallest spacing without electrical contact between the two lines. The electrical contact would indicate a short circuit in the later on printed S-D electrode layer. The most reliable and at the same time the smallest spacing was employed as design rule for the development of the S-D electrode patterns. Further information about the methodology for the S-D electrode finger spacing optimization is found in Supporting Information S3.

### Basic electrical characteristics and variability of the TFTs

**Figure 3:**
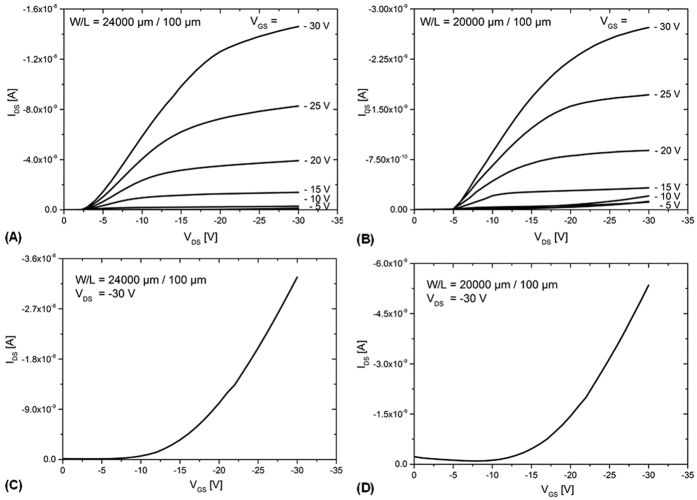
Electrical characteristics of the all-inkjet-printed TFTs showing exemplarily the TFT performance of the best functional devices with (**A**) output curves and (**C**) transfer curve as well as the worst functional device with (**B**) output curves and (**D**) transfer curve.

The performance of the all-inkjet-printed TFTs is shown exemplarily in Fig. 3 based on typical current-voltage (*I*-*V*) or output characteristics and transfer curves measured in saturation region.

Figure 3A,C show the output and the transfer characteristics for one of the best functional TFTs measured (W/L = 240). Fig. 3B,D show the electrical characteristics for a bad performing device (W/L = 200). Both I-V characteristics are highly non-linear for low V_DS_ voltages. Source and drain contacts are not ohmic. This might be caused by the formation of a high resistive area in the vicinity of the drain and source electrodes or a barrier that imped carrier injection. The major difference between the two TFTs is on the on/off ratio. The best TFT shows a significant higher current modulation.

Comparatively, good and bad performing devices do not show dramatic differences in threshold voltage or in field effect mobility. This suggests that the major source of variability is due to variations on the TFT off-current.

**Figure 4:**
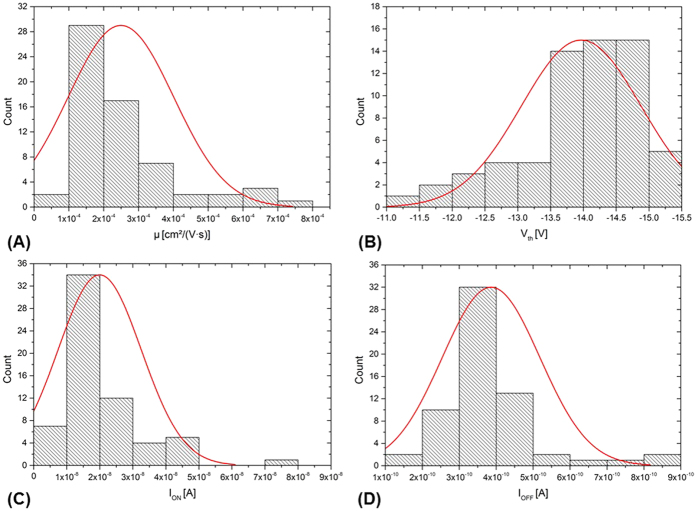
Histograms showing the parameter distribution of the all-inkjet-printed TFTs: (**A**) mobility, (**B**) threshold, (**C**) I_ON_ and (**D**) I_OFF_ distribution.

Figure 4 shows exemplarily the variability of 64 functional TFTs with a W/L of 140 based on histograms of various TFT performance values. Fig. 4A represents the mobility that does not clearly follow the normal distribution curve. It is more close to the Weibull distribution. The mobility in saturation regime is on average 0.00026 ± 0.00015 cm^2^/(V∙s). Thus, the deviations among the devices are quite high. This is mainly caused by inhomogeneities of the OSC layer as a consequence of the printing process (see explanations later on) and by changes of the dimensions of the devices, e.g. the channel length and channel width. The maximum mobility value for W/L of 140 is about 0.00076 cm^2^/(V∙s). The maximum mobility taking in consideration all TFT sizes is 0.00082 cm^2^/(V∙s). Fig. 4B–D represent the distributions of the threshold voltage, I_ON_ current and I_OFF_ current, respectively. Compared to mobility, they are more close to a normal distribution. The threshold voltage is on average about −14.0 ± 0.9 V. The I_ON_/I_OFF_ ratio is on average 55 ± 41. The large distribution of the I_ON_/I_OFF_ ratio is mainly caused by the high variation of the off-current. Reasons for the high off-current are variations of the OSC layer dimensions (e.g. thickness) or layer structure and probably also leakage current variations in the dielectric layer.

Parameter variability can be better visualized by plotting a set of transfer curves for identically printed TFTs with the same dimensions. Fig. S4 in the Supporting Information shows a set of 10 transfer curves. All the transfer curves run parallel to each other. This proves that the variability on field effect mobility itself is small. Thus, the variability is found mainly in the off-current which causes additional dispersion in the threshold voltage.

### TFT manufacturing yield as a function of array position

Figure 2C shows the percentage of functional TFTs per printed array. The yield ranges between 69% in array A2 and only 6% in array C1. In average, the manufacturing yield is about 34%. There is obviously a very high yield difference on the printed DIN A4 substrate between the different arrays. The arrays A1, B1 and C1 have a much lower yield than A2, B2 and C2. The final yield strongly depends on the position of the arrays on the substrate. In the arrays A1, B1 and C1, the printing process starts moving to A2, B2 and C2 and finally moves back and starts again. Thus, A1, B1 and C1 represent the parts that are printed first (before A2, B2 and C2) after a longer period of non-jetting when the printhead is moving back from the end of the substrate to the starting point. This period of non-jetting causes instabilities in the droplet formation process. Some of the droplets are not formed or not formed properly. Thus, the deposition process is disturbed. Droplets might deviate from the targeted position on the substrate due to a quality loss in jet straightness. Several DIN A4 substrates using the same TFT design were manufactured using similar printing parameters to confirm this behavior. We found that the trend is highly reproducible and all the time similar to Fig. 2C. The average yield varied about ± 10%.

We explain this behavior as follows: Volatile ink components will start to evaporate at the nozzle orifice during the non-jetting period. Additionally, the evaporation process will be supported by the heated substrate table causing an increased evaporation rate. This can result in an increase of viscosity and difference in surface tension at the nozzle orifice. However, the energy level of the acoustic pressure waves generated by the piezoelectric transducer of the inkjet printhead that is required for drop ejection depends strongly on the viscosity of the fluid. The higher the viscosity of the fluid, the higher the energy required to eject a droplet and thus usually the higher the driving amplitude of the waveform applied to the piezoelectric printhead. Since the waveform remains constant during the deposition process, the generated acoustic pressure wave might be not able to eject the droplets due to the higher viscosity of the ink at the nozzle orifice when the printhead returns to the idle state at the printing start position. Using nanoparticles in the ink formulation such as for the silver nanoparticle ink might contribute additionally to nozzle failures since the nanoparticles will undergo agglomeration during the solvent evaporation. This causes material buildup in the printhead and especially at the nozzle orifice influencing negatively the drop ejection. The explained phenomena usually termed as “first drop problem” is well known in the field of inkjet printing since many years and has been addressed by several researchers in review articles or detailed experimental studies^22^,^23^,^24^,^25^,^26^,^27^,^28^,^29^,^30^. The length of time for an ink remaining idle at the nozzle orifice of a printhead before droplet ejection is no longer possible is termed as latency^31^. It has been reported, that even for short non-jetting periods of about 1 s a droplet speed degradation can occur for water-based ink formulations^32^. Famili *et al*.^29^ and Verkouteren *et al*.^30^ investigated the first ejected droplets of an inkjet deposition sequence and observed significant differences in terms of droplet shape, trajectory, velocity and volume to those droplets that follow in the sequence. Besides evaporation effects at the nozzle orifice or on the nozzle plate, acoustic and fluid resonances causing acoustic instability in the printhead can contribute to the first drop problem^29^,^30^. Therefore, the first drop problem is a result of a combination of different effects. Nowadays, the first drop problem has been reduced to a minimum for graphic inkjet applications. The inks are usually ready to be printed at all times. The printheads, inks, waveforms applied in jetting and idle state and cleaning procedures are perfectly coordinated with each other and standardized as a result of many years of development and optimization works. However, in the field of functional inkjet printing such as printed electronics, these components and parameters are still needed to be harmonized since they are usually not intentionally designed for each other. The functional fluids for printed electronics are still under development and are quite complex, e.g. due to their chemical and rheological properties combined with nanoparticle ingredients.

### Overview of failures origins causing defective TFTs

**Figure 5:**
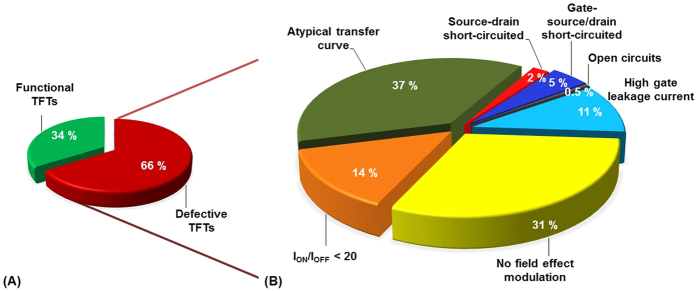
Statistical data of 924 all-inkjet-printed TFTs, (**A**) shows the proportion of functional TFTs to defective TFTs, (**B**) is a pie chart of the 66% defective TFTs presented in (**A**), the defective TFTs are assigned to cause of failure categories as defined in Table 1 (percentage is rounded).

**Figure 6:**
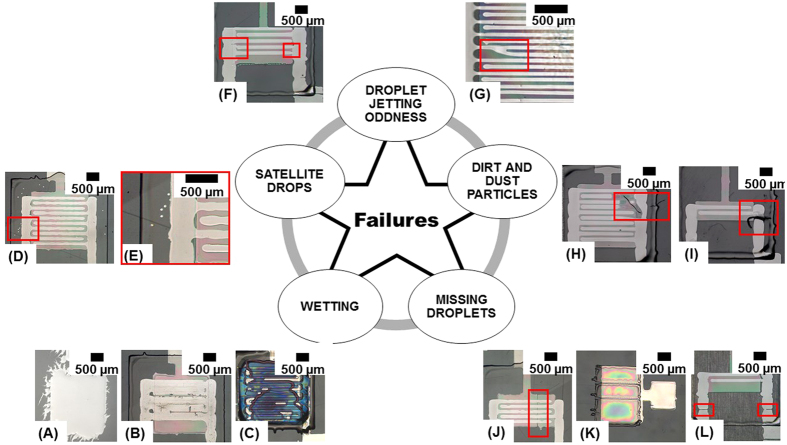
Overview of failures detected in the printed layers of the all-inkjet-printed TFTs, (**A**–**C**) are failures due to wetting properties of the deposited ink on the printed surface: (**A**) depicts a printed gate electrode with intense spreading, (**B**) shows intense ink spreading of the S-D electrodes resulting in a short-circuit and (**C**) depicts inhomogeneous layer formation of the OSC due to the surface energy contrast between cPVP layer and S-D silver electrodes; (**D**) shows satellite drops of silver ink and (**E**) is a magnification of the marked area in (**D**); (**F**,**G**) represent short-circuited S-D electrodes due to odd drop jetting; (**H**) is an example of a short circuit between S-D and (**F**) between drain and gate electrode due to dirt and dust particles appearing during printing or the post-processing; (**J**,**L**) present TFTs with an open circuit due to missing droplets in the gate and the S-D layer, and (**K**) shows the dielectric layer with missing lines due to inkjet nozzle clogging.

In the following section the focus is set on the defective TFTs to study the different kinds of failures. All the TFTs printed on a DIN A4-sized PEN substrate were characterized as described in the materials and methods and assigned to cause of failure categories. Fig. 5 presents the statistical results of 924 all-inkjet-printed TFTs and Fig. 6 provides an overview of the different failures based on microscopic images. As shown in Fig. 5A, about 34% of the TFTs are functional while 66% of the TFTs are classified as defective. Thus, the process yield is comparable low. Fig. 5B shows in detail the causes of the failures and their distribution.

As shown in Fig. 5B, most of the devices (37%) suffer from atypical transfer curves. Atypical transfer curves characterize a non-monotonic behavior and/or negative slopes in the transfer curve. About 31% of the defective devices are classified as devices with a lack in field effect modulation. About 14% of the defects are related to a low current ratio (I_ON_/I_OFF_ < 20). All these failures are operational instabilities that do not necessarily refer to printing problems but to material or contamination issues, e.g. due to the processing in ambient condition enabling the integration of impurities and water molecules in the TFT stack. The operational instabilities are most probably caused by a high density of trap sites and ionic impurities in the bulk of the dielectric and at the interface between dielectric and the OSC (interface states). Once these traps are filled an internal field is established that effectively shields the external applied gate bias and prevents the observation of field effect modulation. cPVP has been frequently used as gate dielectric in literature but it is also well known for its problematic nature based on hydroxyl groups^33^. The hydroxyl groups are responsible for the flexibility of the material and are reactive groups allowing a simple thermal cross-linking. However, a complete cross-linking of the polymer chain of the material is difficult to achieve^34^ – even at a temperature of 150 °C. Usually, hydroxyl groups remain on the surface and in the bulk resulting in polar, hygroscopic properties that act as charge trapping sites. Consequently, electronic in-homogeneities will appear that dramatically influence the device performance. Additionally, water molecules might be absorbed in the dielectric layer during the manufacturing in ambient condition.

Only a very small part of the TFTs have short or open circuits (<8%). The low percentage of S-D short-circuits is a result of the optimization of the spacing between the S-D finger electrodes extracted prior to the TFT manufacturing as explained before (see Supporting Information S3). A comparable conservative design rule was applied resulting in larger channels but limiting the probability of short circuits. Examples for short circuits between the S-D electrodes are shown in Fig. 6F,G. As shown in Fig. 6F, the short circuits can appear between the S-D electrode busbars or directly between the S-D fingers as depicted in Fig. 6G depending on the direction of the jetting oddness. Droplet jetting oddness resulting in droplet placement errors is one of the main reasons for the short circuits. It is preferred that the droplets have to be deposited orthogonally to the nozzle plate in a straight line towards the substrate typically within 10 mrad accuracy^35^. However, in many cases in printed electronics the 10 mrad accuracy cannot be obtained due to several reasons. Droplet jetting oddness is caused e.g. by air bubbles or particle agglomeration in the ink or material build-ups of the ink on the nozzle plate or at the nozzle orifice causing instable droplet break-off. Other influencing factors for jetting oddness are electrostatic charges or aerodynamic effects such as soft draughts resulting from the movement of the printhead, the movement of the droplets or from the exhaust system and printhead defects/nozzle failures in general.

Also, dirt and dust particles are an important cause for short circuits between the S-D electrodes as shown in Fig. 6H where a fiber-like dirt particle connects S-D electrodes due to the intense coalescence of silver ink along the particle. Dirt and dust particles were recognized frequently on the PEN substrate, since all the printing processes were performed in a standard laboratory without high level of cleanliness in ambient condition. Another cause are the wetting problems of the silver ink on the dielectric layer causing an intense spreading of the S-D electrodes and a short circuit as shown in Fig. 6B. The failure origin is the interaction of surface energy of the cPVP layer and surface tension of the silver ink. Furthermore, wetting problems can appear as well for the gate electrode and the OSC layer. Some of the printed silver gate electrodes showed intense, irregular lateral spreading resulting in a low edge sharpness and thus fringing, as demonstrated in Fig. 6A. The image shows bleeding of the silver ink on the PEN substrate. The reason might be the locally high hydrophilicity of the substrate attracting the highly polar silver ink – e.g. due to electrostatic charging. Via optical characterization, we also found several TFTs that were classified as functional with satellite droplets of silver ink as shown in Fig. 6D,E. These occasionally generated extra droplets can have negative impact on the printed layers e.g. forming short circuits. However, in most of the cases we could not see any influence of the satellite drops on the functionality or performance of the TFTs since they were usually located outside the active area.

A defective dielectric layer can result in short circuits between gate and source or/and drain electrodes or to a high gate leakage current. The defective dielectric layer can be caused by several reasons related to the inkjet process, the nature of the dielectric material and its processing. Failures related to the inkjet process are missing droplets due to jetting problems (that finally result in very thin dielectric layers or dielectric layers with missing lines) or as a misalignment of the print layers to each other e.g. due to axis or print layout inaccuracy. Failures related to the nature of the dielectric material and its processing are e.g. problems with the thermal crosslinking process so that the dielectric will be penetrated by the solvent of the on top printed S-D electrodes. Further reasons for defective dielectric layers are dirt and dust particles. Since the printing was performed in ambient condition, dirt and dust particles in the micrometer range are ubiquitous in the ambient air as well as in the printing system. Fig. 6I depicts an example of a TFT with a dirt particle short-circuiting the gate with the drain electrode.

Less than 0.5% of the defective devices have open circuits. The main reason for open circuits are missing lines in the electrode layers due to droplet jetting oddness, missing droplets or dewetting phenomena. Usually, simple nozzle clogging due to solvent evaporation resulting in a complete breakdown of the jetting causes open circuits. Fig. 6J shows a missing line in a printed gate electrode and Fig. 6L shows a TFT with missing lines in the S-D electrodes contact pads. Both were identified as open circuits during the electrical characterization process. The missing lines appeared most frequently for the electrode layers and for only a few devices in the dielectric layer (thus resulting a short circuit). It is much more difficult to identify missing droplets by one or more nozzles generated irregularly in the printed layer. In these cases, it is not possible to see the missing droplets in form of a line in the print pattern as indicated in Fig. 3J–L. A profile of the layer would be required to identify problems such as a locally low layer thickness. For the dielectric layer, the low thickness can result in higher probability to have pinholes resulting in low dielectric resistance, high gate leakage currents under device operation or even short circuits between the gate and source or/and drain electrodes.

### Short circuits and open circuits

**Figure 7:**
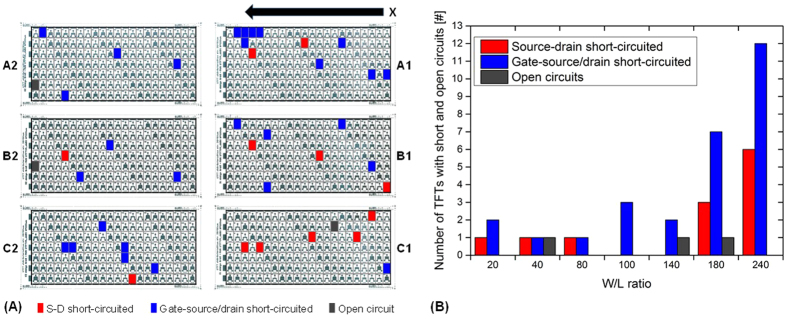
(**A**) Position map of printed TFTs: each color pixel represents the position of a defective TFT: the red pixels indicate TFTs with S-D short circuits, the blue pixels TFTs with gate-source/drain short circuits and grey pixels open circuits; (**B**) histogram based on the position map showing the number of TFTs with short and open circuits as a function of W/L ratio.

Figure 7A shows the position map of the printed DIN A4 substrate with 924 TFTs. The basis of the map is an optical scan using a standard flat-bed office scanner of the DIN A4 substrate with all the printed TFTs. Thus, the printed silver layers appear dark green to black due to the high reflectance of their surface. Next to the TFTs, test areas, alignment marks and other patterns can be seen on the scanned image. These patterns were employed to align accurately the different print layers, to identify the arrays and the individual TFTs and to analyze some standard print layer geometries. The scan also allows to identify the spatial uniform size (W/L ratio) distribution of TFTs. The six arrays are marked with frames and grids has been laid on each of the arrays. Based on the grids, the defective TFTs can be marked by coloration of the respective pixel in the grid. Thus, a colored pixel represents the position of a defective TFT. Here, pixels that are marked red represent TFT having short-circuited S-D electrodes and pixels that are marked blue represent TFT with short-circuited gate-source/drain electrodes. Grey pixels are TFTs with open circuits. Obviously, the arrays A1, B1 and C1 are the most affected areas. Especially in case of the short circuits between the S-D electrode. Thus, the trend is similar to that of Fig. 2C. This distribution is mainly caused by the above mentioned first drop problem. The printhead has jetting failures due to the long idle period to move the printhead back to the print start position. In the first arrays, the jetting process is clearly unstable. However, it seems to recover over the time since the arrays A2, B2 and C2 have less short circuits of S-D electrodes. The distribution of the short circuits between gate-source/drain electrodes is less clear. Many defects appear in array A1. The dielectric ink formulation exhibited a superb droplet jetting process characterized by well-formed droplets and a high process stability (observation was made with the installed drop watcher system). The ink is a solution without any nanoparticles simplifying the inkjet printing process compared to the silver nanoparticle inks applied for the TFT electrodes. Thus, we suppose that most of the short circuits through the dielectric are not caused because of issues related to the jetting process, but due to dirt and dust particles and surface spikes of the silver layer underneath. However, no trend can be derived.

The histogram in Fig. 7B shows the defective TFTs as a function of the TFT size given by the W/L ratio. TFTs with larger W/L ratios are more frequently affected by short circuits than TFTs with smaller W/L ratios. The probability of defects increases with the size of the TFT because larger TFTs have (i) a higher number of finger electrodes increasing the risk of a short circuit between them and have (ii) larger overlapping electrode areas that should be separated by the dielectric increasing the risk of short circuits through the dielectric.

### TFTs with high leakage current

**Figure 8:**
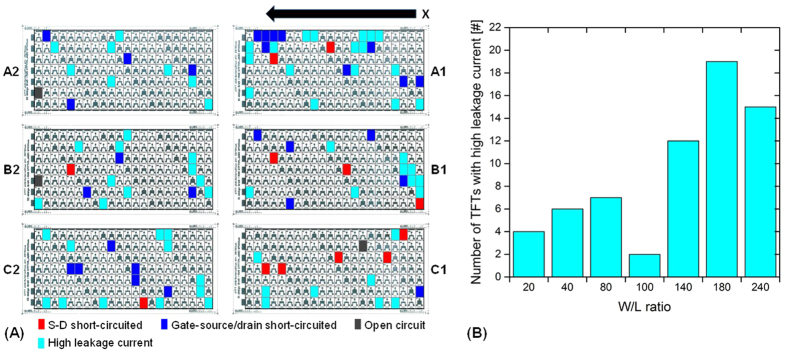
(**A**) Position map of printed TFTs indicating high leakage current devices (cyan pixels); red, blue and grey pixels are defective TFTs determined before; (**B**) histogram based on the position map showing the number of TFTs with high leakage current as a function of W/L ratio.

Figure 8A shows a position map of TFTs with high leakage current (cyan pixels). Red pixels indicate the before determined defective devices having short-circuited S-D electrodes, blue pixels the devices with short-circuited gate-source/drain electrodes and grey pixels open circuits. The failures in Fig. 8A are not in all cases clearly correlated to the printing area. However, in total TFTs with smaller area (W/L ratio) are less affected by high leakage currents. Since the jetting process of the dielectric ink formulation was characterized by well-formed droplets and a high process stability, we suppose that most of the TFTs with high leakage current through the dielectric are caused by dirt and dust particles, wetting problems and surface irregularities of the silver layer underneath.

**Figure 9:**
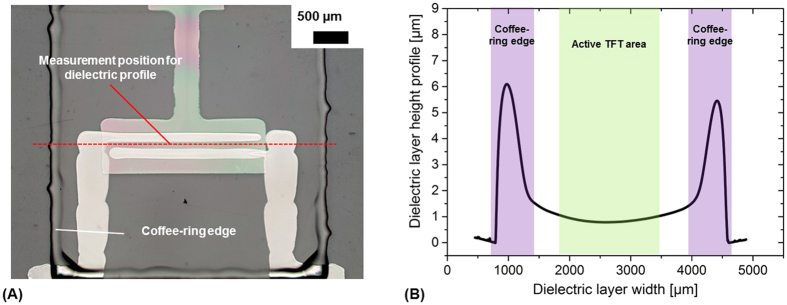
(**A**) Printed layer stack of gate electrode, dielectric layer and S-D electrodes; the dielectric layer has a remarkable coffee-ring edge designed far outside the active area of the TFT; (**B**) height profile of the dielectric layer determined along the center of the TFT as indicated in (**A**).

The number of devices with high leakage current is reasonable low. The dielectric layer was optimized during the manufacturing several times. The aim was to facilitate the formation of a smooth and homogeneous dielectric surface. However, inkjet printing of a thin, pinhole-free dielectric layer with smooth surface characteristics and high uniformity is a challenge. We found that thin dielectric layers of about 1 μm thickness with homogeneous surface morphology were obtained in the center of the TFT by exploiting the well-known coffee-ring effect^36^. Since the edge of the dielectric layer with peak heights of more than 5 μm is clearly shaped by the coffee-ring effect as shown in Fig. 9, the dielectric layer area was designed comparable large so that the layer edge is far away from the active area of the TFT. Taking advantage of the coffee-ring effect that transports most of the dielectric solute to the edge of the layer, a thin and homogeneous dielectric film was formed reliably in the center of the TFT as indicated in Fig. 9B. In addition to profilometry, FIB cutting was employed to obtain a cross-sectional view of the dielectric layer. Based on spot checks at the different TFT arrays on the substrate, the dielectric thickness is about 1080 ± 190 nm. We could see a slight drift of thickness by comparing the arrays A1, B1 and C1 with A2, B2 and C2. A2, B2 and C2 have in average slightly higher dielectric thickness (about 10%). Representative SEM images showing the cross-sectional areas of the printed TFTs are provides in the Supporting Information (Fig. S5)

### TFTs with low field effect modulation

**Figure 10:**
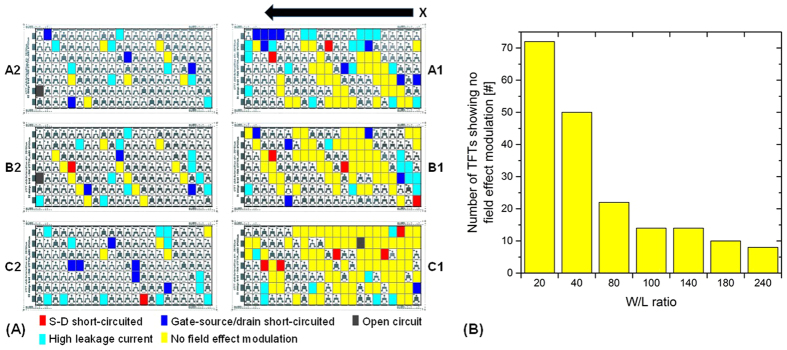
(**A**) Position map indicating TFTs having no field effect modulation colored as yellow pixels; the other colors represent defective TFT determined in test procedures before; (**B**) histogram based on the position map depicting the number of TFTs showing no field effect modulation as a function of W/L ratio.

About 31% of the all-inkjet-printed TFTs have low or no field effect modulation (see Fig. 5B). The percentage is much higher than the total sum of short-circuits, open circuits, and high leakage current failures. Fig. 10A shows the distribution of the printed TFTs that show no field effect modulation colored with yellow pixels over the substrate and Fig. 10B is a histogram of the defective devices as a function of TFT size. The defects are directly related to the array areas and to the TFT size.

**Figure 11:**
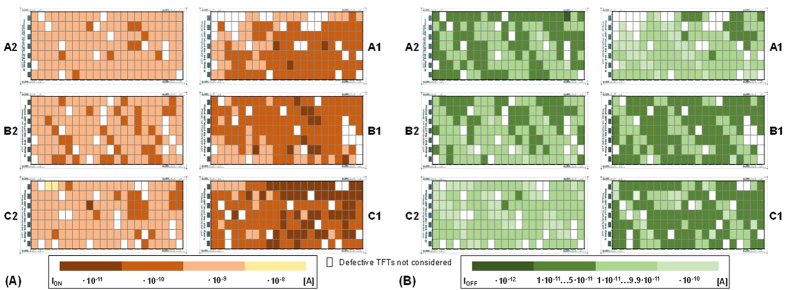
Position maps of normalized (**A**) I_ON_ and normalized (**B**) I_OFF_ values over the substrate; white pixels are all the defective TFTs with short circuits, open circuits or high leakage current.

Again, most of the defective devices appeared in arrays A1, B1 and C1. Additionally, this type of failure occurs frequently in small area TFTs having low W/L ratio. In the arrays A2, B2 and C2, most of the smaller TFTs (W/L ratio of 20) do not have field-effect modulation as indicated in the position map of Fig. 10A. Therefore, the TFT size has a direct relation with the phenomena that degrades the field effect modulation. Fig. 11 shows a position map indicating the normalized I_ON_ (V_DS_ = −30 V and V_GS_ = −30 V) and I_OFF_ (V_DS_ = −30 V and V_GS_ = 0 V) values of the TFTs. Obviously, there is a strong gradient in the I_ON_ and I_OFF_ values over the substrate. The I_ON_ shows a clear trend: The TFTs in the arrays A1, B1 and C1 have much smaller currents than the TFTs in A2, B2 and C2. The position map of the I_ON_ data shows similarities to the map of Fig. 10A. There is a clear relationship between I_ON_ and TFTs having no field effect modulation (e.g. compare array B2 in Figs 10A and 11A). Again, many TFTs with low W/L ratio have comparable low I_ON_ values. In average, I_OFF_ is about 0.59 pA ± 3.3 pA Most of the values are in the range of 0.1 pA to 0.99 pA as shown in Fig. 11B. Thus, the spread is less for the values of I_OFF_ than for the values of I_ON_.

In order to investigate the reason for the distribution of the current, SEM analysis was performed on the TFT samples treated with FIB to obtain a cross-sectional view on the layer stack. The thickness of the OSC layer of the TFTs located on A2, B2 and C2 was determined to about 500 nm to 700 nm while the thickness of the OSC on the arrays A1, B1 and C1 was in average about 100 nm to 400 nm (see also Supporting Information Fig. S5). Therefore, as expected the thicker the OSC layer the higher the I_ON_ values.

We explain the difference in layer thickness of the OSC between the TFT arrays mainly based on the first drop problem. The first drop problem was substantially supported by the substrate temperature during the printing process which was set to 70 °C and the temperature of the nozzle plate set at 30 °C. 70 °C substrate temperature was required to avoid de-wetting of the OSC ink on the layer stack. Lower temperature increased the risk for the formation of non-continuous OSC layers as shown in Fig. 6C. 30 °C temperature of the nozzle plate was required to stabilize the ink conditions since the printhead and ink warm up during the printing process over the substrate with elevated temperatures. Some of nozzles potentially clog on the way back to the start position when the printhead is in idle mode. During printing of the array A1, B1 and C1, the jetting process was disturbed such that the deposited OSC volume was dramatically reduced. Compared to other cases in literature, the duration of the disturbance in our example seems to be unusual long for the first drop problem. Obviously, the problem remains for at least half of the A4-sized substrate length which sums up to several thousands of droplets. It might be a very extreme example of the first drop problem due to the high temperature of the substrate and the relatively large area of printing. Without heating the substrate or the ink, a droplet of mesitylene of about 10 pL volume has a calculated lifetime of about half a second^37^. Considering the temperature applied in our process, the evaporation will be much faster so that it seems quite reasonable to assume that ink starts to dry already at the nozzle orifice resulting in jetting instability. The reduction of deposited materials from the arrays A1, B1 and C1 to A2, B2 and C2 leads finally to very thin OSC layers with high resistance on the A2, B2 and C2. As a consequence, many of the printed TFTs were rated as defective during the electrical analysis. Thinner OSC layers are more permeable to the diffusion of atmospheric species such as H_2_0 which can migrate to the dielectric surface contributing to an increase of the density of deep traps. This effect also explains why TFTs with small areas are more prone to fail^38^. Small area TFTs show thinner OSC thickness. Therefore, thick semiconductor layers are desirable because they act as protective encapsulation preventing the contamination by atmospheric agents.

### TFTs with atypical transfer curve or/and I_ON_/I_OFF_ < 20

**Figure 12:**
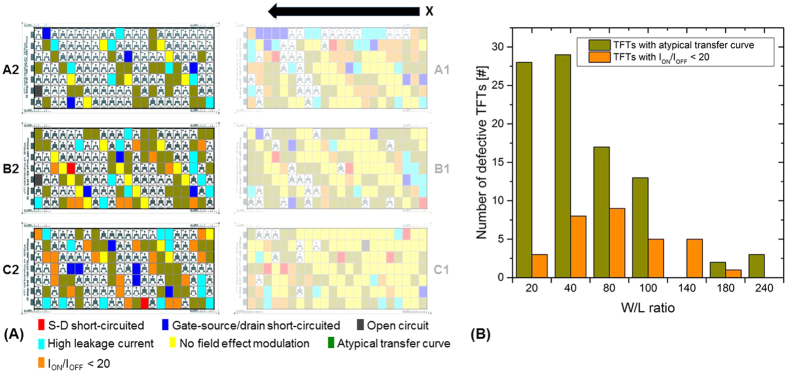
(**A**) Position map indicating TFTs with atypical transfer curve and I_ON_/I_OFF_ < 20 colored as green and orange pixels; (**B**) histogram based on the position map showing the number of defective TFTs classified in low I_ON_/I_OFF_ ratio and devices having an atypical transfer curve.

Due to the high quantity of failures that already appeared in the arrays A1, B1 and C1, only the arrays A2, B2 and C2 were considered for the analysis of the stability test to increase the statistical relevance. Fig. 12A shows the failure map and Fig. 12B the histogram. A1, B1 and C1 are marked blurry since they were not considered for the histogram data as explained before. Green pixels represent TFTs with an atypical transfer curve such as curves with non-monotonic behavior and/or negative slope. Orange pixels represent TFTs with I_ON_/I_OFF_ < 20. Pixels marked with other colors indicate the TFTs sorted out in the test procedures before. Although A1, B1 and C1 are marked blurry, it can be seen that most of the defects appeared again in these arrays. This seems to be reasonable due to the mentioned first drop problem and in general fault propagation. TFTs of smaller size thus having lower W/L ratio show a more unstable behavior in the transfer curve similar to the trend in Fig. 10A due to gate-bias stress issues and the explained high electrical resistance of the OSC layer due to the low thickness. The size distribution of TFTs with low I_ON_/I_OFF_ ratio is not very clear. No trend can be derived. However, taking also into account the arrays A1, B1 and C1, the trend would be quite similar as the one for the TFTs with atypical transfer curves confirming as assumed above that both types of failures have the same physical origin.

### Summarized distribution of failure origins of defective TFTs

**Figure 13:**
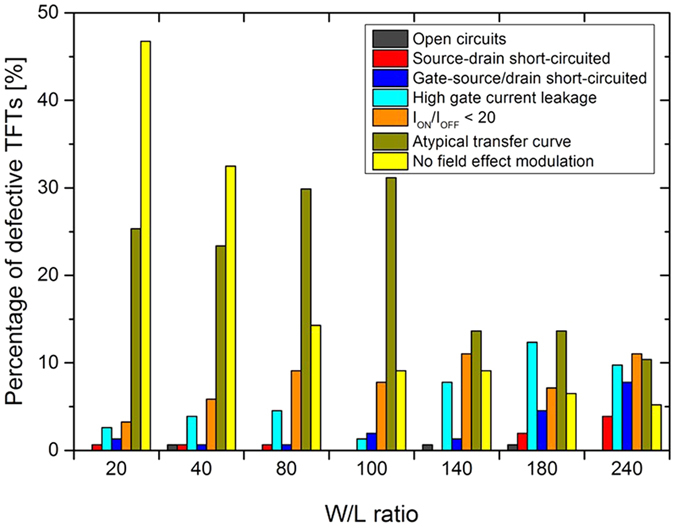
Summary of device failure distribution as a function of W/L ratio, 610 defective TFTs out of the 924 all-inkjet-printed TFTs were considered in this graph.

Figure 13 shows the device failure distribution of the defective TFTs as a function of TFT size represented by the W/L ratio. The trends for the different origin of device failures are clearly visible. Open circuits are related to unconnected silver tracks which appeared only three times in case of 924 printed TFTs. Therefore, the influence of this failure is very low and no statistical relevant data about the failure distribution can be derived. The number of short-circuited TFTs increase with the W/L ratio for both S-D short circuits as well as short circuits between gate and S-D. This is reasonable since the TFT area increases with increasing W/L ratio and thus more droplets and finger electrodes are printed increasing the risk of short circuits, e.g. due to ink coalescence or defects in the dielectric as presented in Fig. 6H,I,K. TFTs with high gate leakage current and I_ON_/I_OFF_ < 20 follow similar trend. The higher the W/L ratio, the higher the number of TFTs affected.

Figure 13 shows that the smaller the TFT the higher is the number of failures caused by lack of field effect modulation or atypical transfer curves. This trend is not so systematic for the failure caused by atypical transfer curves. However, it is clear that smaller TFTs are more affected than TFTs having larger areas.

## Summary and Conclusion

TFT arrays were manufactured entirely using inkjet printing technology in ambient conditions. A detailed electrical characterization of the manufactured TFTs was performed focusing on the defective devices. The aim was to identify and to study the different categories of defects and their origins. We found several types of defects directly related to the inkjet printing process such as open circuits caused by missing lines e.g. due to nozzle clogging or short circuits due to odd droplet jetting. However, the first drop problem was identified as a major cause of device defects. There is a clear dependency of failure distribution over the substrate as a function of position of the individual TFTs as well as a function of TFT size. Mostly, the arrays close to the print start position are affected by failures. Improvement of the jetting is required and the implementation of jetting failure detection technologies^39^,^40^ to allow a more stable and reliable inkjet manufacturing process for printed electronics application.

We also identified defects such as short-circuits due to dirt and dust particles or defects resulting in operational instabilities that do not relate directly to the inkjet printing process. These defects are related to the contaminations, impurities and the nature of the used materials. Our contribution demonstrates the detailed identification of failures and their dependence on the TFT size for all-inkjet-printed devices and thus provides important information for future process optimization towards higher manufacturing yields and industrial application.

## Materials and Methods

### Substrate and inks

Polyethylene naphthalate (PEN) films (Dupont Teijin Q65FA) with a thickness of 125 μm were employed as flexible polymeric substrate. Silver nanoparticle ink from Sun Chemical (SunTronic SunJet Silver EMD5603) was applied for the manufacturing of the Gate and Source/Drain (S-D) electrodes. The ink was filtered with a 0.45 μm syringe filter with GHP membrane (Pall) and treated ultrasonically for 15 minutes to remove or re-disperse larger nanoparticles or agglomerations. Finally, the ink was degassed before printing at 180 mbar for 10 minutes. Cross-linked poly-4-vinylphenol (cPVP) was applied as dielectric material. Poly-4-vinylphenol (PVP) was dissolved in 10 mL of propylene glycol monomethyl ether acetate (PGMEA) at room temperature supported by magnetic stirring for 3 hours. Poly(melamine-co-formaldehyde) methylated (PMFM, from Aldrich, Mn about 432, 84 wt% in 1-butanol) was added as a crosslinking agent and the final solution was stirred for 12 hours. The weight ratio of PVP to PMFM was 5:1. Before printing, the PVP-based dielectric solution was diluted in PGMEA (volume ratio 1:1) and filtered with a 0.2 μm syringe filter to remove residual agglomerations. FS0096, a p-type polymer from Flexink (Flexink Ltd., UK) which is dissolved in mesitylene was used as organic semiconductor (OSC).

### Fabrication of TFTs

A Dimatix Materials Printer 3000 (DMP3000, Fujifilm Dimatix, USA) was employed for the deposition of the silver nanoparticles ink, dielectric ink and OSC ink. It was equipped with a Fujifilm Dimatix SE3 printhead having 128 nozzles arranged in a single line with a nozzle size of 42 μm and a nominal drop volume of 35 pL for the silver nanoparticle ink and the dielectric ink. For the OSC (formulation FS0096), the DMP3000 was equipped with a laboratory cartridge printhead with 16 nozzles each with a diameter of about 21.5 μm and 10 pL nominal drop volume in order to obtain thin OSC layers. For all the layers, the printing process was carried out with a maximum jetting frequency of 5 kHz.

**Figure 14:**
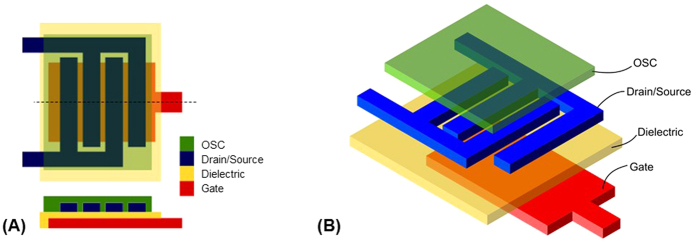
(**A**) Layout of the design of the TFTs as side and top view, (**B**) 3D representation of the BGBC TFT structure.

The chosen TFT architecture was the BGBC (Bottom Gate Bottom Contact) design to avoid the OSC material suffering from thermal curing processes of silver and dielectric layers needed in a TGBC (Top Gate Bottom Contact) architecture. The TFT comprises of (from bottom to top): a bottom gate layer (silver nanoparticle ink), an insulating dielectric layer (cPVP), S-D electrodes (silver nanoparticle ink) and a p-type OSC (FS0096) as shown Fig. 14.

Prior to printing, the substrate PEN was cleaned with ethanol, and then dried by a nitrogen flow to remove any remaining particles. For the deposition of the bottom gate, the silver ink was jetted using a print resolution of 635 dpi and 27 nozzles. The post-printing procedure consisted of drying and sintering at 135 °C for 30 minutes. Then, two layers of cPVP were printed using a print resolution of 635 dpi and 42 nozzles. After the printing of the first dielectric layer, the sample was dried on the table of the printer at 40 °C for 10 minutes, and afterwards the second layer was printed and dried on the printer table at 40 °C for 10 minutes similar to the previously printed first layer. Final curing and crosslinking of the dielectric layer took place at 150 °C for 45 minutes. For the S-D electrodes, silver ink was printed directly onto cPVP. The pattern was deposited using 508 dpi and 11 nozzles. The layer was sintered at 135 °C for 30 minutes. Finally, the OSC film was printed using 1270 dpi and 9 nozzles. The post-printing procedure for this layer consisted of a simple drying at 100 °C for 20 min. Post-processing of all the deposited thin-films was done in oven. To control the layer formation, the printer table was heated for the deposition of silver ink to about 50 °C, for the dielectric ink to about 40 °C and for the OSC to about 70 °C. For the deposition of the OSC, the printhead was heated to 30 °C to ensure stable conditions during printing and proper layer formation. For the deposition of the S-D electrodes on top of the cPVP the cartridge of the printhead was heated to 50 °C. The samples were stored in a low vacuum system in dark conditions. All processing and characterization steps were carried out in ambient condition. Further information about the manufacturing process is available elsewhere^17^.

### TFT Print layout

A total number of 924 BGBC TFTs with different channel width (W) to channel length (L) ratios (W/L, ratio of 20, 40, 80, 100, 140, 180 and 240) were designed as six arrays (A1, A2, B1, B2, C1, C2) to allow better electrical characterization on A4-sized PEN substrate using the EDA (Electronic Design Automation) software Clewin (WieWeb Software Inc., Netherlands). The geometrical dimensions of the TFTs are provided in the Supporting Information (Table S1). The TFTs are spatially uniformly distributed to avoid failures into the same group of TFTs per row due to printing effects affecting the yield statistics. Their arrangement in the six arrays is identical.

A scheme of the layout is shown in Fig. 1A. The unidirectional printing direction is indicated in Fig. 1A with a black arrow and the printing origin is marked with x. Further information about the printing direction is available in Supporting Information (Fig. S1). The influence of the printing direction on morphological properties of deposited lines was studied elsewhere^20^. As depicted in the Fig. 1B, the print pattern layout consists of four separated layers: (i) gate, (ii) dielectric, (iii) S-D electrodes, and (iv) OSC. The area of the dielectric layer was kept constant in order to have identical layer forming conditions and thus identical morphological layer characteristics for all the TFTs.

The final device is shown in Fig. 1D. The left image depicts the TFT without the OSC in order to observe clearly the gate, dielectric and S-D electrodes. The image on the right in Fig. 1D shows the TFT including the OSC layer. Fig. 1C shows the all-inkjet-printed A4-sized PEN substrate with 924 TFTs.

### Instrumentation

The optical microscope Leica DM 4000 M was used to analyze the printed layers. Electrical measurements were carried out in a semi-automatic Cascade Microtech Summit Series 12000 probe station using a semiconductor parameter analyzer Keithley 4200 and taking into account the IEEE 1620 standard for test methods for the characterization of organic transistors and materials^41^. The probe station was adapted to work with the soft and flexible plastic substrate. Different smart characterization protocols were developed based on LabVIEW programs which perform a variety of electrical measurements in order to analyze the failure origins. Additionally, *in-situ* optical investigation was performed by capturing microscopic images of each of the TFTs during the process of electrical characterization. Profilometry measurements were carried out with a Veeco Dektak 150. Scanning electron microscopy (SEM) was performed with a Zeiss Auriga microscope. The system is equipped with a focused ion beam (FIB) tool Zeiss 1560XB Cross Beam. FIB cuts were performed to obtain cross-sectional images of the layer stack in order to determine the thickness and the structure of the individual layers. The cuts were 150 μm long to avoid single point measurements in non-regular areas and were done in the center of the TFTs far away from the edge regions.

### Methodology for the failure detection and failure categories

Thousands of TFTs were analyzed. Device failures were detected by means of a semi-automatic electrical characterization platform. The electrical characterization was preferred over other morphological contact-scanning methods or optical methods since (i) the layers are deposited on flexible substrates, (ii) layer thicknesses are in the nanometer or low micrometer range and (iii) some of the layers are transparent or semitransparent posing a challenge for some of the optical-based methods. Thus, the electrical characterization was considered as more reliable, efficient and less time consuming than optical methods. Furthermore, electrical methods are extremely sensitive to the presence of electrical active impurities which affect device performance and their ability to work properly as a transistor. However, spot checks with optical microscopy as well as profilometry and SEM were performed as well to confirm the structural or morphological nature of some of the defects.

TFTs fail to operate properly due to several types of malfunctions. A detailed overview of the failures and their origins is shown in Table 1 and will be discussed in the following sections. The detected TFT failures were classified into two main types. The first type is caused by physical defective regions such as short circuits or interrupted electrodes resulting in open circuits between any of the TFT terminals. These defective regions are related to failures during the printing process such as missing nozzles, wetting issues of the ink, dirt and dust particles and other environmental impacts. The second type of malfunction is related to operational instabilities of the TFT. This type of failure is related with the electronic process and includes two types of phenomena: (i) build-up of internal electrical fields caused by charge carrier trap filling, (ii) tunneling currents or soft-dielectric breakdown across thinner dielectric regions leading to leakage current. Although, leakage current can be also a result of physical defective regions our analysis shows that defective regions such as pinholes are present in a reduced number and they do not contribute to the statistical analysis. The leakage currents across the dielectric might be tolerable to a certain extend and do not result into total device failure.

Anomalous or atypical transfer curves are curves that show a non-monotonic behavior or/and negative slope. Successive measurements of these transfer curves lead to a progressive degradation of the I_ON_/I_OFF_ ratio. After resting for long periods (two days) the device may recover the original behavior. This type of failure was attributed to a fast trap filling mechanism and therefore included in the class of operational instabilities. We believe that the poor field effect modulation is intimated related with the same electronic process (trapping) that also causes the atypical transfer curves. Therefore, devices with a I_ON_/I_OFF_ ratio < 20 were also classified in the failure type named “operational instabilities”.

## Additional Information

**How to cite this article**: Sowade, E. *et al*. All-inkjet-printed thin-film transistors: manufacturing process reliability by root cause analysis. *Sci. Rep.* **6**, 33490; doi: 10.1038/srep33490 (2016).

## Supplementary Material

Supplementary Information

## Figures and Tables

**Table 1:**
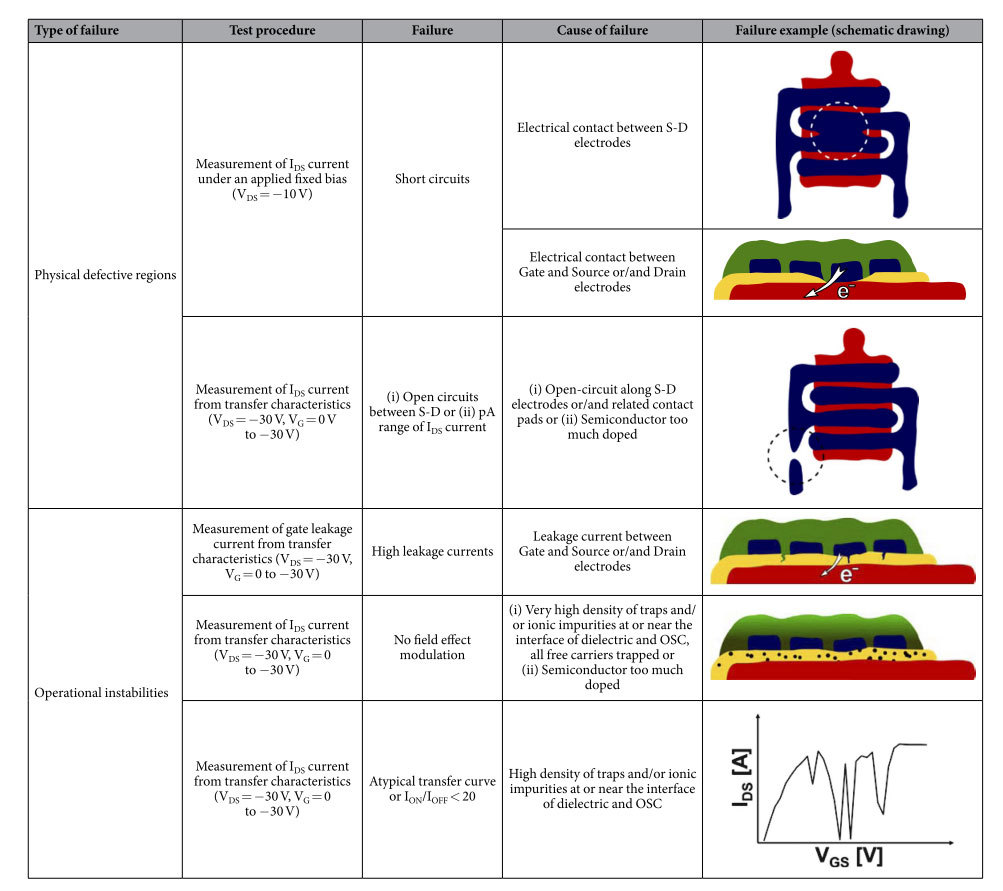
Overview of electrical tests, failures and failure origins of all-inkjet-printed TFTs.

Further detailed information about the developed electrical characterization process including a measurement flow chart is available in the Supporting Information (see in Supporting Information S2 and Fig. S2).
