# Supplementary material for: All-inkjet-printed thin-film transistors: manufacturing process reliability by root cause analysis
Source: Sci Rep. 2016 Sep 21;6:33490. doi: 10.1038/srep33490 (PMC5030703; doi:10.1038/srep33490)
Supplement: Supplementary Information [file srep33490-s1.pdf]

All-inkjet-printed thin-film transistors: manufacturing process reliability by root cause analysis

Enrico Sowade1*#, Eloi Ramon2#, Kalyan Yoti Mitra1, Carme Martinez-Domingo2, Marta Pedró2, , Jofre Pallarès2, Fausta Loffredo4, Fulvia Villani4, Henrique L. Gomes5,6, Lluis Teres2, Reinhard R. Baumann1,7

1 Technische Universität Chemnitz (TUC), Digital Printing and Imaging Technology, 09126 Chemnitz, Germany.

2 Institut de Microelectrònica de Barcelona, IMB-CNM (CSIC), 08193 Bellaterra, Spain

4 Italian National Agency for New Technologies, Energy and Sustainable Economic Development (ENEA), Portici Research Center, 80055 Portici (Naples), Italy

5 Universidade do Algarve, FCT, Campus de Gambelas, 8000-139 Faro, Portugal

6 Instituto de Telecomunicações (IT), Organic Electronics - Lx, 1049 - 001 Lisboa, Portugal

7 Fraunhofer Institute for Electronic Nanosystems (ENAS), Department of Printed Functionalities, 09126 Chemnitz, Germany.

* corresponding author: phone +49.(0)371.531.38326; fax +49.(0)371.531.8.38326; e-mail enrico.sowade@mb.tu-chemnitz.de

# Authors contributed equally to the presented work

Table S1: Geometrical dimensions of the designed TFTs

W/L	W [µm]	L [µm]	a*b [µm²]	c*d [µm²]	e [µm]	f [µm]	g [µm²]	h*i [µm²]
**20**	2000	100	2200 x 650	3700 x 3750	2000	150	50	2600 x 850
**40**	4000	100	2200 x 850	3700 x 3750	2000	150	50	2600 x 1050
**80**	8000	100	2200 x 1250	3700 x 3750	2000	150	50	2600 x 1450
**100**	10000	100	2200 x 1450	3700 x 3750	2000	150	50	2600 x 1650
**140**	14000	100	2200 x 1850	3700 x 3750	2000	150	50	2600 x 2050
**180**	18000	100	2200 x 2250	3700 x 3750	2000	150	50	2600 x 2450
**240**	24000	100	2200 x 2850	3700 x 3750	2000	150	50	2600 x 3050

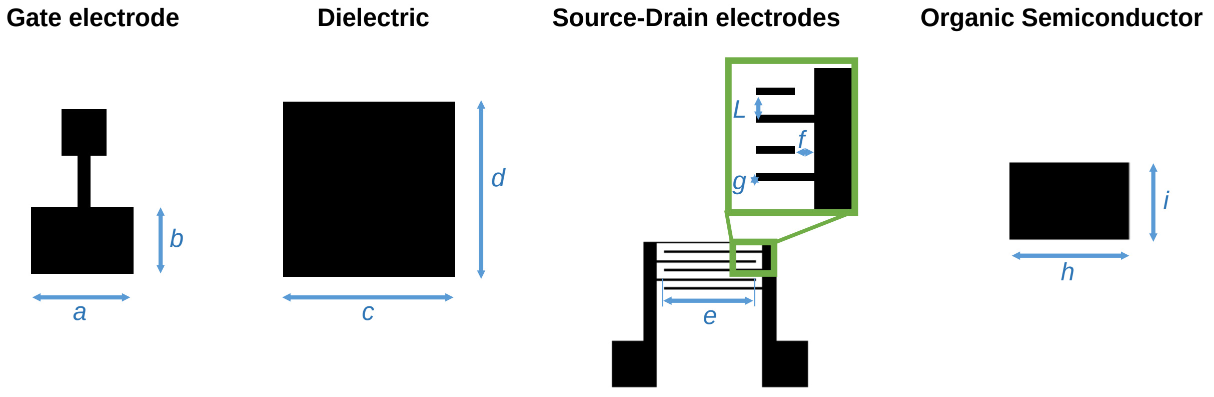

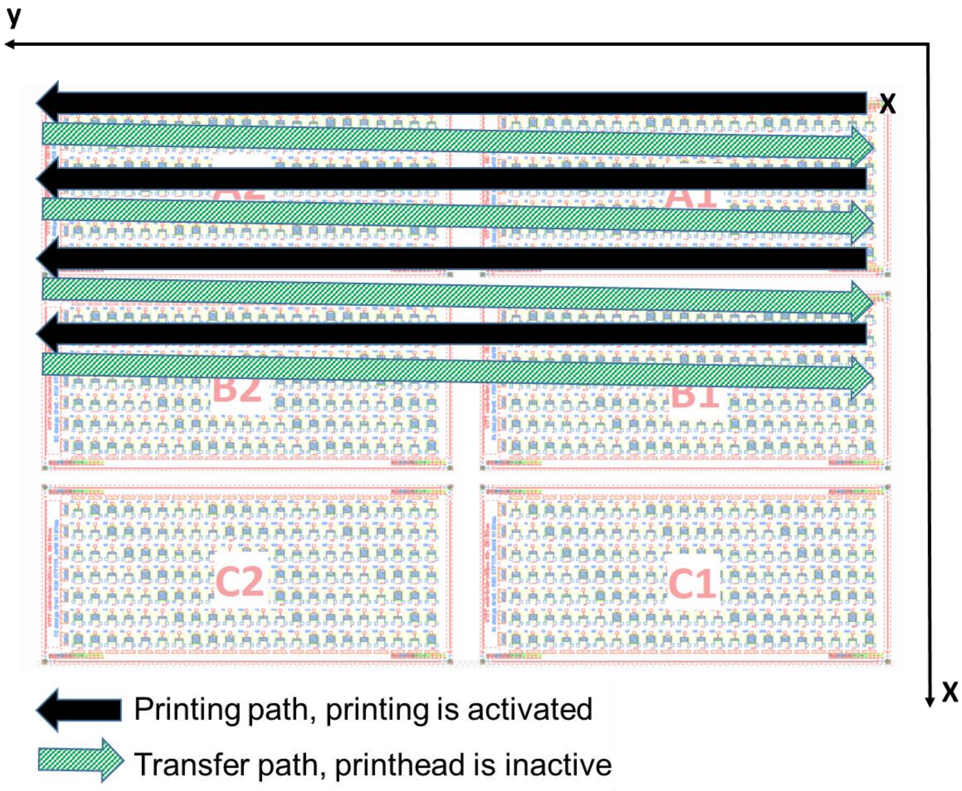

Figure S1: Process description of the inkjet printing procedure divided in a printing path and a transfer path; the inkjet deposition is active only for the printing path and idle for the transport part resulting in a unidirectional deposition process

S2: Methodology and process flow of the electrical characterization

Two main phases are performed in the electrical characterization test flow. The first phase is based on the extraction of electrical parameters biasing the three terminals of the TFT at certain fixed voltages. The purpose of this part is to discard failures such as short- and open-circuits not related to the performance of the device by means of threshold values for different electrical parameters. This measurement is useful since TFT can be discarded in advance before applying more time consuming measurements for the extraction of the transfer curve. The second phase obtains the amount and position of functional devices through the analysis of the transfer curve. Following flow diagram (Figure S2) indicates the applied characterization tests and their parameters.

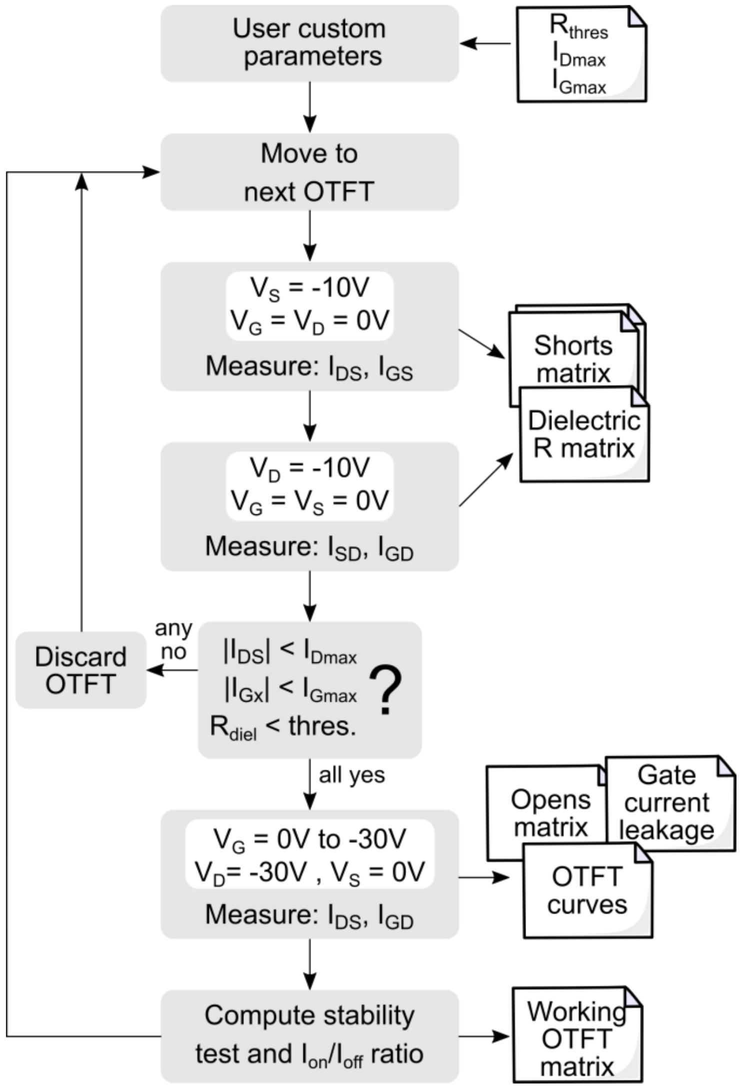

Figure S2: Flow chart of the electrical characterization procedure of the all-inkjet-printed TFTs

First electrical test phase

In the first test phase, the source electrode is biased at -10 V whereas gate and Drain electrodes were grounded. I_{DS} (D-S current) and I_{GS} (gate-source current) parameters are determined. Then, the drain electrode is biased at -10 V whereas gate and source electrodes are grounded. I_{SD} (S-D current) and I_{GD} (gate-drain current) parameters are measured. The low voltage applied in this first part was chosen to prevent damages of the dielectric layer, e.g. resulting in initial electrical breakdowns through the gate dielectric.

Short circuits due to printing issues between the S-D electrodes were detected in case of absolute I_{DS} or absolute I_{SD} parameters exceeding a current threshold I_{Dmax} of 10 mA. Based on the threshold test, a excel file with a matrix of short circuits between S-D is automatically generated mapping the amount and position of TFTs with and without short circuits. In addition, two matrixes are generated for short-circuits between drain-gate terminals and Source-Gate terminals indicating if the TFT passes or fails in case of I_{GD} and I_{GS} do exceed the current threshold I_{Gmax} of 10 mA. Afterwards, the resistance of the gate dielectric for each TFT is calculated. Considering only the highest absolute values of I_{GS} or I_{GD} currents, a dielectric resistance matrix in a excel file is generated indicating if the resistance is lower than a threshold R_{diel} of 1 GΩ. The failure origin for this matrix is basically due to printing issues during the dielectric layer. Thus, if the results of the measured values do not exceed the threshold for each parameter, the TFT is further characterized by the second test phase.

Second electrical test phase

In the second test phase, the transfer curve in linear region is extracted for those TFTs that have passed the first test phase. A sweep from 0 V to -30 V is applied to the gate and the S-D electrodes are biased at -30 V and 0 V, respectively. I_{DS} and I_{GD} curves are extracted. TFT transfer curves are plotted from I_{DS} and gate voltage values. Using the same test procedure, open circuits of the S-D electrodes can be detected. In comparison with the first test phase where I_{DS} is measured at -10 V, a much higher voltage of -30 V is applied enabling the measurement of sufficient current intensity to differentiate between an open circuit and functional devices. The current for an open circuit or no field effect modulation in the TFT is usually in the range of nano- to pico ampere and the I_{DS} current of functional devices is usually proportional to the resistance of the OSC. Finally, a excel file is generated mapping the open circuit matrix for TFTs with $I_{DS} < 1$ nA. For this case, the nature between open circuit failures due to printing issues of S-D or the intrinsic properties of the semiconductor leading to no field effect modulation failures can not be distinguished. Moreover, the I_{GD} value at -30 V is compared to the I_{DS} value at the same voltage. In case of $I_{GD} > I_{DS}$, the TFT is classified into the high leakage current category and thus marked as defective.

Although some of the TFTs pass all the previous tests, not all of them have a good electrical and TFT-like behavior. From the transfer curve, a stability test is applied which consist of two conditions for accepting a printed TFT as functional: (i) the ratio of I_{on}/I_{off} > 20 and (ii) the transfer curves shows a monotonic behavior with a positive slope. I_{on} is the drain current at -30 V and I_{off} is the drain current at 0 V. As a result of the transfer curve test, a final excel file is generated mapping the functional TFTs matrix. The manufacturing yield can also be determined.

S3: Methodology for S-D finger electrode spacing optimization

The distance between the S-D finger electrodes depends strongly on the ink and substrate/layer surface interaction, e.g. the surface tension of the ink and the surface energy of the substrate or the already deposited layer. As demonstrated, also the printing direction and thus printing process parameters influence the distance between the S-D electrode fingers. Specific design rules are required allowing the deposition of optimized S-D electrodes and the design rules are a function of the specific fabrication process including used inks and substrates, the precision and resolution of the printing machinery, post-processing treatments such as sintering and many more.

The inkjet process requires the formalization of the required design rules for designing the different layers of the TFT. The S-D electrode layer is one of the most important layers since the design is quite complex due to the interdigitated finger architecture. A set of parameterisable cells (called Pcells) was used to generate test matrixes of lines with different separation distances (called test vehicles) simulating the S-D finger electrodes. These PCells were printed with the DMP3000 system using defined printing parameters and based on the printout, the required rule values were extracted by mapping short-circuited and non short-circuited test vehicles. Figure S3A shows such as printed PCell sample. Figure S3B and S3C are examples of different separation distance. The separation distance between the electrodes in Figure S3B is too low and result in a short circuit. In contrast, the separation distance between the electrodes in Figure S3C is such

such separated electrodes appear without any short circuit. The separation distance was varied and hundrets of samples were printed for each distance to determine the most reliable but at the same times lowest separation distance to be applied for the S-D electrodes in the TFT manufacturing process.

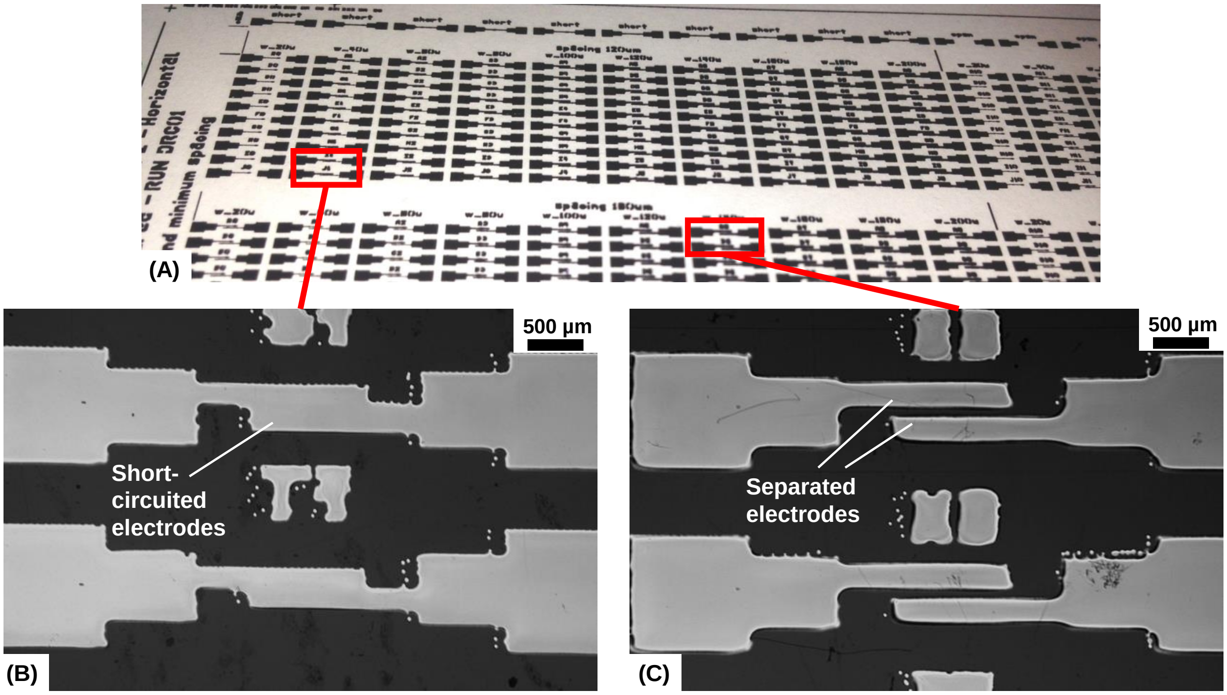

Figure S3: (A) Inkjet-printed test layout (Pcells) consisting of test vehicles designed as lines with different spacing between themselves, (B) and (C) are microscopic images showing exemplarily a section of (A) with (B) short-circuited silver electrodes due to a very low separation distance in the design layout and (C) well-separated silver electrodes due to higher separation distance in the design layout

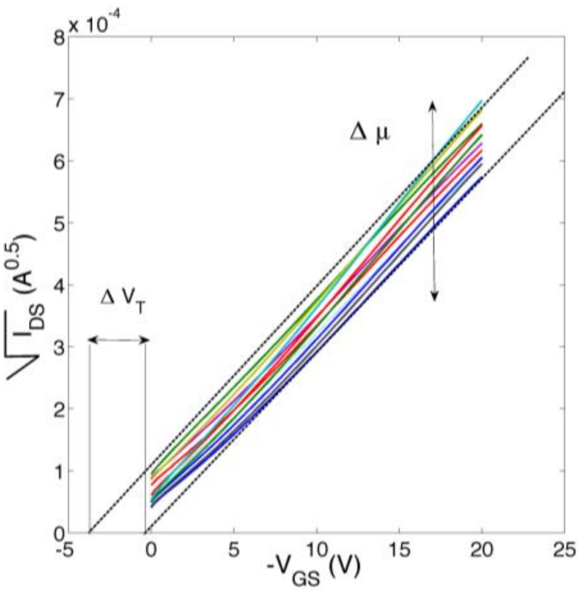

Figure S4: Set of transfer curves measured in the saturation region (V_{DS} = - 20 V) for 10 TFTs printed with identical process parameters

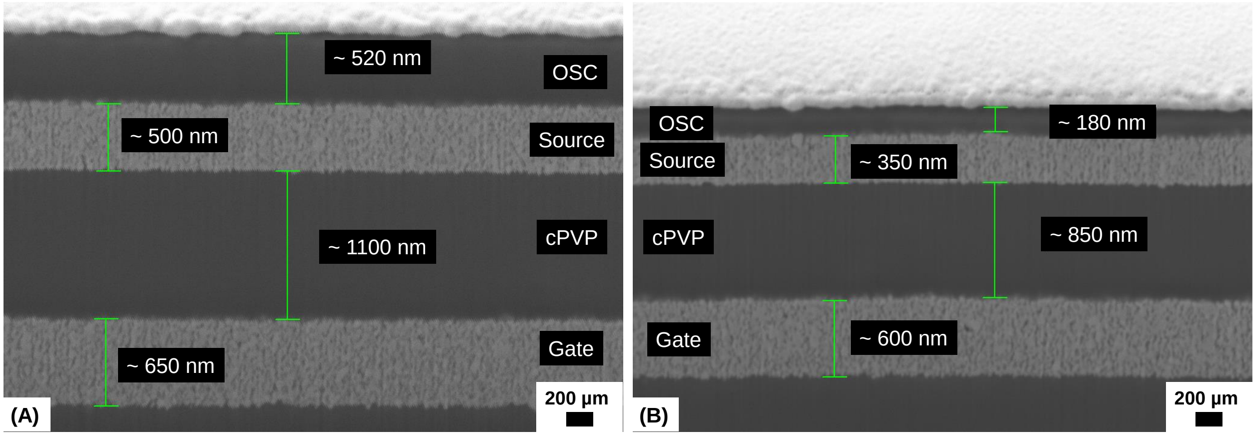

Figure S5: SEM images showing a cross-sectional view of the printed TFTs, (A) is a TFT of the array A2 and (B) a TFT of the array A1; various of these spot-checks were performed and similar thicknesses were obtained, the layers in the arrays A1, B1 and C1 have usually lower thicknesses compared to the layers in A2, B2 and C2 as indicated in the images
